# Differential Effects of Munc18s on Multiple Degranulation-Relevant Trans-SNARE Complexes

**DOI:** 10.1371/journal.pone.0138683

**Published:** 2015-09-18

**Authors:** Hao Xu, Matthew Grant Arnold, Sushmitha Vijay Kumar

**Affiliations:** Department of Biological Sciences, University of Southern Mississippi, Hattiesburg, Mississippi, United States of America; Institute of Molecular and Cell Biology, Biopolis, UNITED STATES

## Abstract

Mast cell exocytosis, which includes compound degranulation and vesicle-associated piecemeal degranulation, requires multiple Q- and R- SNAREs. It is not clear how these SNAREs pair to form functional trans-SNARE complexes and how these trans-SNARE complexes are selectively regulated for fusion. Here we undertake a comprehensive examination of the capacity of two Q-SNARE subcomplexes (syntaxin3/SNAP-23 and syntaxin4/SNAP-23) to form fusogenic trans-SNARE complexes with each of the four granule-borne R-SNAREs (VAMP2, 3, 7, 8). We report the identification of at least six distinct trans-SNARE complexes under enhanced tethering conditions: i) VAMP2/syntaxin3/SNAP-23, ii) VAMP2/syntaxin4/SNAP-23, iii) VAMP3/syntaxin3/SNAP-23, iv) VAMP3/syntaxin4/SNAP-23, v) VAMP8/syntaxin3/SNAP-23, and vi) VAMP8/syntaxin4/SNAP-23. We show for the first time that Munc18a operates synergistically with SNAP-23-based non-neuronal SNARE complexes (i to iv) in lipid mixing, in contrast to Munc18b and c, which exhibit no positive effect on any SNARE combination tested. Pre-incubation with Munc18a renders the SNARE-dependent fusion reactions insensitive to the otherwise inhibitory R-SNARE cytoplasmic domains, suggesting a protective role of Munc18a for its cognate SNAREs. Our findings substantiate the recently discovered but unexpected requirement for Munc18a in mast cell exocytosis, and implicate post-translational modifications in Munc18b/c activation.

## Introduction

Mast cells play critical roles in immunity and allergic inflammation through regulated release of various biologically active mediators (e.g., histamine, proteases, and cytokines) [1]. Many of these mediators are pre-stored in secretory lysosomes/granules that, upon mast cell activation, undergo signaling-dependent compound degranulation (homotypic fusion with one another and heterotypic fusion to the plasma membrane) [2] or piecemeal degranulation (granule-associated cargos are packaged into small vesicles that subsequently transport to and fuse with the cell surface) [3]. Like all fusion events along the endocytic and secretory pathways, mast cell exocytosis requires SNAREs {SNAP [soluble NSF (N-ethylmaleimide-sensitive factor) attachment protein] receptors} anchored to apposed membranes to form a fusogenic 4-helical bundle, the so-called trans-SNARE complex [4]. A functional or fusogenic trans-SNARE complex is typically formed by three Q-SNAREs (Qa, b, c) emanating from one membrane and one R-SNARE from the other [4]. However, for mast cell exocytosis, the nature of the underlying trans-SNARE complex(es) has been elusive, in part due to the presence of quite a number of degranulation-relevant SNAREs [5,6,7,8,9,10,11,12,13].

Localization studies of resting murine mast cells indicate that secretory lysosomes/granules are decorated with R-SNAREs VAMP2, 3, 7, 8 [6,7,12,13,14,15] and Qa-SNARE syntaxin3 [7,12,13,14], whereas the plasma membrane is enriched with Qa-SNARE syntaxin4 [13,16] and Qb,c-SNARE SNAP-23 [6,13]. During mast cell compound degranulation, syntaxin3 relocates from the secretory lysosomes to the plasma membrane [9], whereas SNAP-23 relocates from the plasma membrane to the secretory lysosomes [13]. According to the 3Q: 1R rule [17], these seven SNAREs can potentially form eight distinct trans-SNARE complexes. Among them, VAMP8 (R)/syntaxin4 (Qa)/SNAP-23 (Qbc) represents best established trans-SNARE complex for mast cell degranulation [18], supported by degranulation assays involving knockout mice, primary cells and cultured cell lines [5,6,7,10,11,13,15,19], biochemical characterizations [7,10,19,20] and reconstitution studies [21,22]. However, multiple trans-SNARE complexes are required in activated mast cells to fulfill a number of roles. First, the homotypic and heterotypic fusion in degranulation may each exploit a distinct trans-SNARE complex. A particularly promising candidate for the homotypic granule fusion is syntaxin3, which appears to be the only known Qa-SNARE on the secretory lysosomes. It effectively binds SNAP-23 and VAMP8 [20] and is critical for optimal secretion of β-hexosaminidase in RBL (rat basophilic leukemia)-2H3 cells, a tumor analog of mucosal mast cells [9]. Second, piecemeal degranulation may play a prominent role under conditions where compound exocytosis is compromised and might exploit R-SNAREs other than VAMP8, as suggested by the lack of complete inhibition of β-hexosaminidase release in VAMP8-knockout mast cells [7,15]. In accordance with this, increasing amounts of VAMP2 and VAMP3 were found in association with SNAP-23 in VAMP8 deficient cells [7,8], suggesting a VAMP2- or VAMP3- based trans-SNARE complex might account for the remaining secretory activity. Third, there is heterogeneity within the secretory lysosome population in mast cells [23]. A recent study using mast cells derived from VAMP8-knockout mice showed that while VAMP8 is required for the regulated release of β-hexosaminidase and serotonin, it is dispensable for TNF-alpha and histamine secretion [15]. Thus, VAMP8-independent trans-SNARE complex(es) must exist for regulated degranulation in these cells. A leading alternative to VAMP8, besides VAMP2 and VAMP3, is VAMP7, which has been shown to mediate granule exocytosis in mature human mast cells [10].

The involvement of multiple exocytic trans-SNARE complexes in mast cell is in line with the observation that all three mammalian Munc18 isoforms specific for regulated exocytosis are linked to mast cell degranulation. Munc18s are members of the conserved Sec1-Munc18 (SM) protein family that regulate fusion by exploiting different modes of association with the fusion machinery [24,25]. Noted for its ability to activate the neuronal trans-SNARE complex [26], Munc18a has been thought to function mainly in synaptic transmission, but it is also expressed in non-neuronal tissues [27,28,29]. Very recently, a double knockdown of Munc18a and Munc18b in RBL cells was found to eliminate β-hexosaminidase release, whereas reintroducing Munc18a alone fully rescued the secretion defect [30]. However, through which set of SNAREs Munc18a operates in mast cell exocytosis is not clear. The functional requirement for Munc18b in mast cell exocytosis has long been recognized [16,31], but it was recently that its participation in microtubule-dependent granule translocation, a stage preceding granule fusion, was delineated [9]. Whether Munc18b acts exclusively in translocation during mast cell degranulation or multitasks in both translocation and fusion awaits further clarification. Munc18c is ubiquitously expressed in mammals and interacts with syntaxin4 in a wide range of cells including RBL cells [31]. Although both negative and positive roles of Munc18c in SNARE-mediated GLUT4 exocytosis have been observed [32,33,34,35] [36], the functional importance of Munc18c in mast cell exocytosis remains to be established.

In this study, we used reconstitution to explore the functional pairing of each of the four granule-borne R-SNAREs (VAMP2, 3, 7, 8) with Q-SNAREs syntaxin3, syntaxin4, and SNAP-23. We then investigated the effects of three Munc18 isoforms respectively in each of the reconstituted fusion reactions. We report that Munc18a selectively promotes the lipid mixing mediated by VAMP2 and VAMP3, in a fashion that prevents the inhibitory action of R-SNARE cytoplasmic domains.

## Materials and Methods

### cDNA Constructs

The cDNA encoding rat SNAP-23 in the pGEX vector (gift from Paul Roche) was amplified using PCR and inserted in between the NcoI and EcoRI sites of the pMBP-parallel1 vector [37] to generate pMBP-TCS (TEV Cleavable Site)-SNAP23. Similarly, pMBP-TCS-Syx3 (rat), pMBP-TCS-Syx4 (rat), pMBP-TCS-VAMP2 (rat), pMBP-TCS-VAMP2CD (rat) pMBP-TCS-VAMP3 (rat), pMBP-TCS-VAMP8 (rat) and pMBP-TCS-VAMP8CD (rat) were generated respectively from pGEX-syntaxin3 (gift from Reinhard Jahn), UB339 (Syx4 construct; gift from Ulrich Blank), pGEX-Syb1-116 (gift from Jose Rizo), rat reference cDNA (for VAMP3; Zyagen), and pGEX-KG-endobrevin (gift from Reinhard Jahn). These constructs contain a 4-aa-long linker sequence (GAMG) between the TCS and the start codon of each SNARE for efficient cleavage by TEV, except the VAMP8 constructs which contain a 2-aa-long linker (GA). The cDNA for VAMP7 was amplified from rat reference cDNA and inserted into the LIC site of pET MBP His_6_ LIC cloning vector (gift from Scott Gradia; Addgene plasmid # 37237) to generate pET-VAMP7-TCS-MBP-His_6_. The cDNA for rat Munc18a was purchased from ThermoScientific (Clone ID #7315868), amplified using PCR and inserted in between the EcoRI and SalI sites of pMBP-parallel1 to generate pMBP-TCS-Munc18a. Rat Munc18b cDNA was generated from pCMV-Munc18-2 (gift from Thomas Südhof) and the rat reference cDNA using overlapping PCR to mitigate a point mutation near the 3’ end in pCMV-Munc18-2 and an insertion mutation near the 5’ end in the reference cDNA. The PCR product containing the correct sequence of Munc18b was initially insert into the EcoRI/SalI sites of pMBP-parallel1 for bacterial expression but later subcloned into the BamHI/SalI sites of pFAST-BAC-HT-JS (gift from Jingshi Shen) for insect cell line expression. All the cDNA constructs above were verified via DNA sequencing. Sequences of cloning primers are in S1 Table. pET28a-NSF (hamster) and pET28a- αSNAP (cow) were kind gifts from Reinhard Jahn. pFL-38His_6_-TEV was a kind gift from William Wickner. pFAST-BAC-HT-JS-Munc18c (mouse), pET28a-syntaxin4 and pET15b-SNAP23 were kind gifts from Dr. Jingshi Shen).

### Proteins

All recombinant proteins purified in this study were quantified using the Bradford assay (BioRad) according to manufacturer’s instruction, snap-frozen in small aliquots in liquid N_2_, and stored at– 70°C. To purify MBP-tagged SNAREs, *E*. *coli* Rosetta2 (Novagen) transformed with the respective plasmid was inoculated into 100 mL LB medium containing 100μg/mL ampicillin and 25μg/mL chloramphenicol. Following overnight growth at 37°C, the culture was added to 1L of Terrific Broth (TB) medium containing 100μg/mL ampicillin and 25μg/mL chloramphenicol, and shaken at 37°C until the OD_600_ ≈ 1.5. IPTG (1M) was added to a final concentration of 0.5mM. After 4 hours of continual growth at 37°C, bacteria were harvested by centrifugation (5k rpm, 5 min, room temperature, GS3 rotor). The pellet was resuspended in 20 mL of Buffer A (50mM HEPES-KOH, pH 7.5, 0.5M KCl, 10% glycerol, 1mM DTT) containing 5mM benzamidine, 1mM PMSF, 1x PIC (0.62μg/mL leupeptin, 4μg/mL pepstatin A, and 24.4μg/mL pefabloc-SC), and subject to two passes through a French Press at 900 psi. Except for SNAP-23, VAMP2cd and VAMP8cd, one-tenth volume of 1M n-octyl-β-D-glucoside (β-OG; Affymetrix) was added and incubation was continued at 4°C for 1 h with nutation. The lysates were then centrifuged at 40 k rpm for 1 h in a Beckman 70Ti rotor. The supernatants were added to 4mL of amylose resin (NEB) pre-equilibrated with the wash buffer (50mM HEPES-KOH, pH 7.5, 0.3M KCl, 10% glycerol and 0.1M β-OG). Following 1 h nutation at 4°C, the resin was packed into an empty Biorad Econo-column at 4°C, washed with 40mL of wash buffer, and eluted with 10mM maltose in wash buffer. MBP-SNAP-23, MBP-VAMP2cd and MBP-VAMP8cd were purified in the same fashion except without introducing β-OG in the wash and elution buffer. To purify untagged syntaxin4 and His_6_-SNAP-23, BL21(DE3) co-transformed with pET28a-syntaxin4 and pET15b-SNAP23 was induced with 0.5mM IPTG as described above. The cell pellet from 1L of culture was resuspended in 20mL of Buffer B (25mM HEPES-KOH, pH 7.5, 0.4M KCl, 10% glycerol, 20mM imidazole and 2mM β-mercaptoethanol) containing 5mM benzamidine, 1mM PMSF, 1x PIC, and subject to two passes through a French Press at 900 psi. Following detergent treatment and ultracentrifugation (as above), the supernatant was applied to 3mL of Ni-NTA resin (Qiagen) pre-equilibrated with Buffer B containing 100mM β-OG, and nutated for 2 h at 4°C. Resins were then washed with 30mL of Buffer B containing 100mM β-OG. Proteins were eluted in 25mM HEPES-KOH, pH 7.5, 0.4M KCl, 10% glycerol, 200mM imidazole and 100mM β-OG.

To purify MBP-Munc18a, Rosetta2/pMBP-TCS-Munc18a was grown in 1L of TB at 37°C and induced with 0.2mM IPTG at OD_600_ ≈ 1.0. The culture was subsequently incubated at 22°C overnight before cell pellets were harvested by centrifugation (5k rpm, 5 min, RT, GS3 rotor). Cell pellet was resuspended in 20mL of Buffer C (50mM Tris-Cl, pH 8.0, 500mM KCl, 5mM EDTA) containing 5mM benzamidine, 1mM PMSF and 1x PIC, and then passed twice through a French Press at 900 psi. The Supernatants were collected through ultracentrifugation with a Beckman Type 70 Ti rotor (4°C, 1 hr, 40,000 rpm) and applied to 4mL of amylose resin pre-equilibrated with Buffer C. Following nutation at 4°C for 2 h, the amylose resin was washed with 20mL of Buffer C, then eluted with 20mL of Buffer C containing 10mM maltose. MBP-Munc18a was dialyzed 1,000,000-fold in RB150 (20mM HEPES-NaOH, pH 7.4, 150mM NaCl, 10% glycerol) overnight at 4°C. His_6_-Munc18b and His_6_-Munc18c were affinity-purified using Ni-NTA resin from lysates of transfected Sf9 cells (gift from Fengwei Bai) according to the published procedures [36]. His_6_-Munc18b was dialyzed 27783-fold in RB150, and His_6_- Munc18c was dialyzed 27783-fold into RB150 containing 0.5mM DTT. Proteins were concentrated to desired concentration using 30k MWCO Microsep™ Advanced Centrifugal Device (Pall Corporation) before storage.

Recombinant His_6_-NSF, His_6_-αSNAP and His_6_-tev were each expressed in Rosetta2(DE3) in 1L of Terrific Broth at 37°C. Following addition of 0.5mM IPTG at OD_600_ = 1.2, the cultures were incubated for 4 h before cell pellets were harvested by centrifugation. His_6_-NSF cell pellets were resuspended in 20mL of Buffer D (50mM HEPES-KOH, pH 7.6, 100mM KCl, 0.5mM MgCl_2_, 0.5mM ATP, 5% glycerol, 1mM DTT) containing 10mM imidazole and 1x PIC, whereas His_6_-αSNAP and His_6_-Tev cell pellets were resuspended in 20mL of Buffer A containing 10mM imidazole and 1x PIC. Follow French Press and ultracentrifugation (as above), supernatants were applied to 4mL of Ni-NTA resin (pre-equilibrated with Buffer D or A, each containing 10mM imidazole), and nutated for 2 h at 4°C. After the resins were washed with 20mL of Buffer D or Buffer A (each containing 20mM imidazole), proteins were eluted in the respective buffers that contain 200mM imidazole. Prior to storage, His_6_-αSNAP and His_6_-Tev were dialyzed 1,000,000-fold in RB150 overnight at 4°C, whereas His_6_-NSF was concentrated using 30k MWCO Microsep™ Advanced Centrifugal Device.

### Proteoliposome Preparation

All the fluorescent lipids were obtained from Invitrogen whereas the non-fluorescent lipids were from Avanti Polar Lipids, Inc. Unless otherwise specified, donor proteoliposomes contain 60% POPC (1-palmitoyl-2-oleoyl-sn-glycero-3-phosphocholine), 17% POPE (1-palmitoyl-2-oleoyl-sn-glycero-3-phosphoethanolamine), 10% DOPS (1,2-dioleoyl-sn-glycero-3-phosphoserine), 10% cholesterol, 1.5% NBD-DHPE [N-(7-Nitrobenz-2-Oxa-1,3-Diazol-4-yl)-1,2-Dihexadecanoyl-sn-Glycero-3-Phosphoethanolamine] and 1.5% rhodamine DHPE (Lissamine™ Rhodamine B 1,2-Dihexadecanoyl-*sn*-Glycero-3-Phosphoethanolamine), and acceptor proteoliposomes contain 60% POPC, 19% POPE, 10% DOPS or POPS, 10% cholesterol and 1% Dansyl DHPE [N-(5-Dimethylaminonaphthalene-1-Sulfonyl)-1,2-Dihexadecanoyl-sn-Glycero-3-Phosphoethanolamine]. Proteoliposomes were prepared by detergent dilution and isolated on a Histodenz density gradient flotation as previously described [38]. SNARE proteins were kept at similar densities as other reconstitution studies [26], with protein: lipid rations at or below 1:200 for R-SNARE-bearing donor RPLs (reconstituted proteoliposomes) and at or below 1:500 for Q-SNARE-bearing acceptor RPLs. His_6_-tev was added at 60μg/mL in each reconstitution to remove the N-terminal tags [38].

### Lipid-Mixing Assay

Unless otherwise specified, a standard fusion reaction (20μL) contained R-SNARE donor RPLs (50μM lipids) and Q-SNARE acceptor RPLs (400μM lipids) in RB150. Reactions performed in the presence of NSF/αSNAP also included 0.5mM MgCl_2_, 0.5mM ATP, and an ATP regenerating system (0.5mg/mL creatine kinase and 14.5mM creatine phosphate). Wherever Munc18s were used, the N-terminal MBP or His_6_ tag was removed by premixing the chimeric Munc18s with His_6_-Tev at a molar ratio of 2:1. To monitor lipid mixing, reaction mixtures (prepared on ice, incubated overnight at 4°C or on ice) were transferred to a 396 well plate and the NBD fluorescent signal was measured (λ_ex_ = 460 nm, λ_em_ = 538 nm, λ_cutoff_ = 515 nm) in a SpectraMAX Gemini XPS plate reader (Molecular Devices) at 37°C. The maximal, early rate of dequenching was calculated as the increased fluorescence at any time divided by the fluorescence at the first minute [(F_t_—F_0_)/F_0_]. An increase of 1 in this parameter is defined as one unit. For donor RPLs, the dequenching [(F_d_ /F_0_)-1] in the presence of 2% (v/v) Triton X-100 was around 6 units in this study. To compare two sets of data, dequenching units from multiple repeats of each experimental condition were imported pair-wise into KaleidaGraph 3.6, where p values were calculated using Student’s t test.

## Results

### Seven SNAREs Implicated in Mast Cell Exocytosis Form Multiple Fusogenic Trans-SNARE Complexes

**Fig 1:**
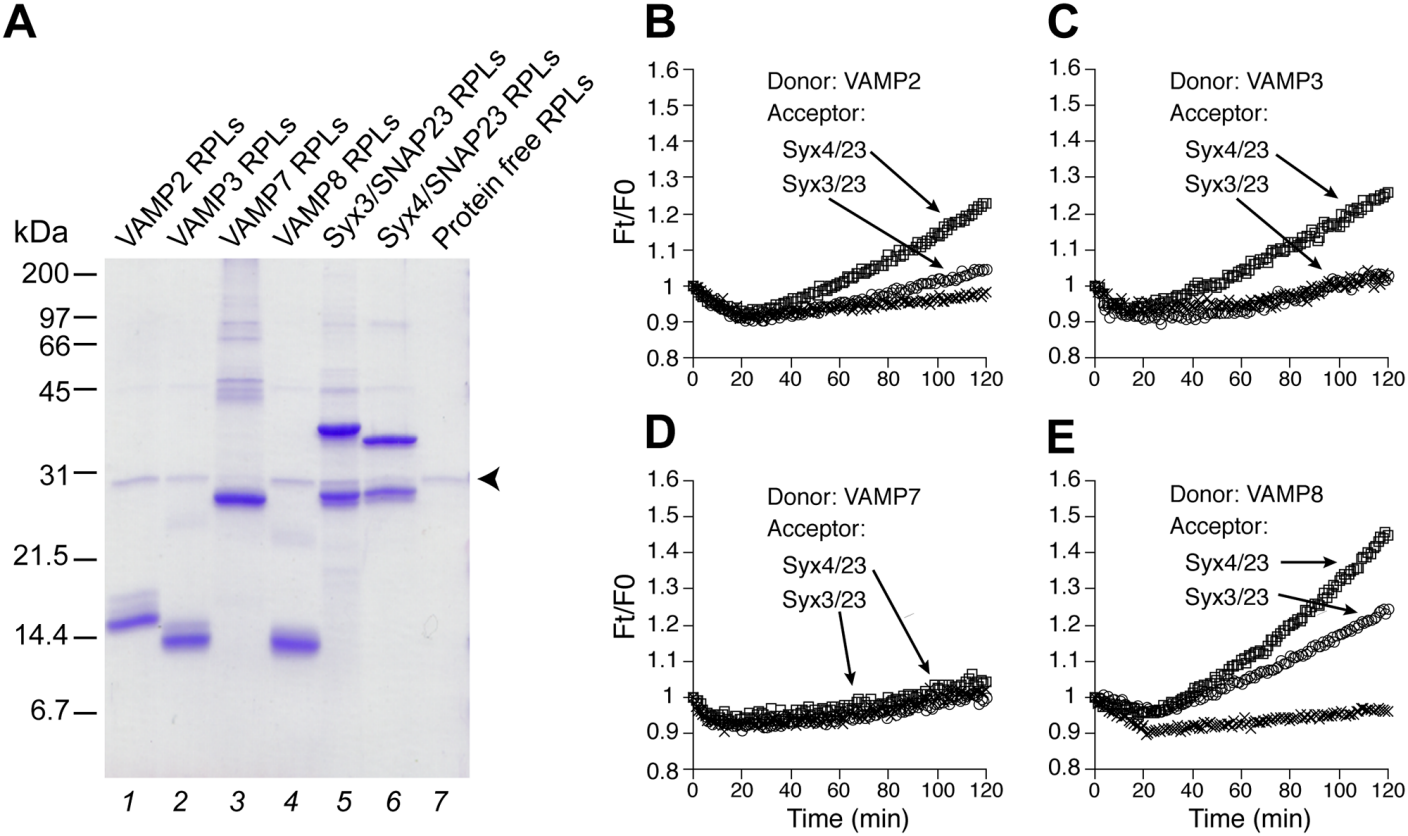
Reconstituted proteoliposomes (RPLs) bearing R- and Q- SNAREs involved in mast cell exocytosis. (A) Coomassie blue-stained SDS-PAG of reconstituted proteoliposomes. A total of 20 nmol (based on total lipids) of donor RPLs (lanes 1 to 4) and acceptor RPLs (lanes 5 and 6) were used in each lane. Small amounts of His_6_-Tev used in the reconstitution get incorporated as well (specified by the arrowhead). The positions of protein markers are indicated on the left. (B to E) Standard fusion reactions. The fluorescence of NBD-DHPE reconstituted in the donor RPLs was measured every min and the dequenching of NBD-DHPE fluorescence (due to lipid mixing) is presented as Ft/F_0_, with Ft being the NBD-DHPE fluorescence at any time point and F_0_ being the fluorescence at the first minute. Represented by x are controls (not readily visible in C and D), in which donor RPLs were incubated with the SNARE-free acceptor RPLs. A representative result from more than three biological replicates is shown.

**Fig 2:**
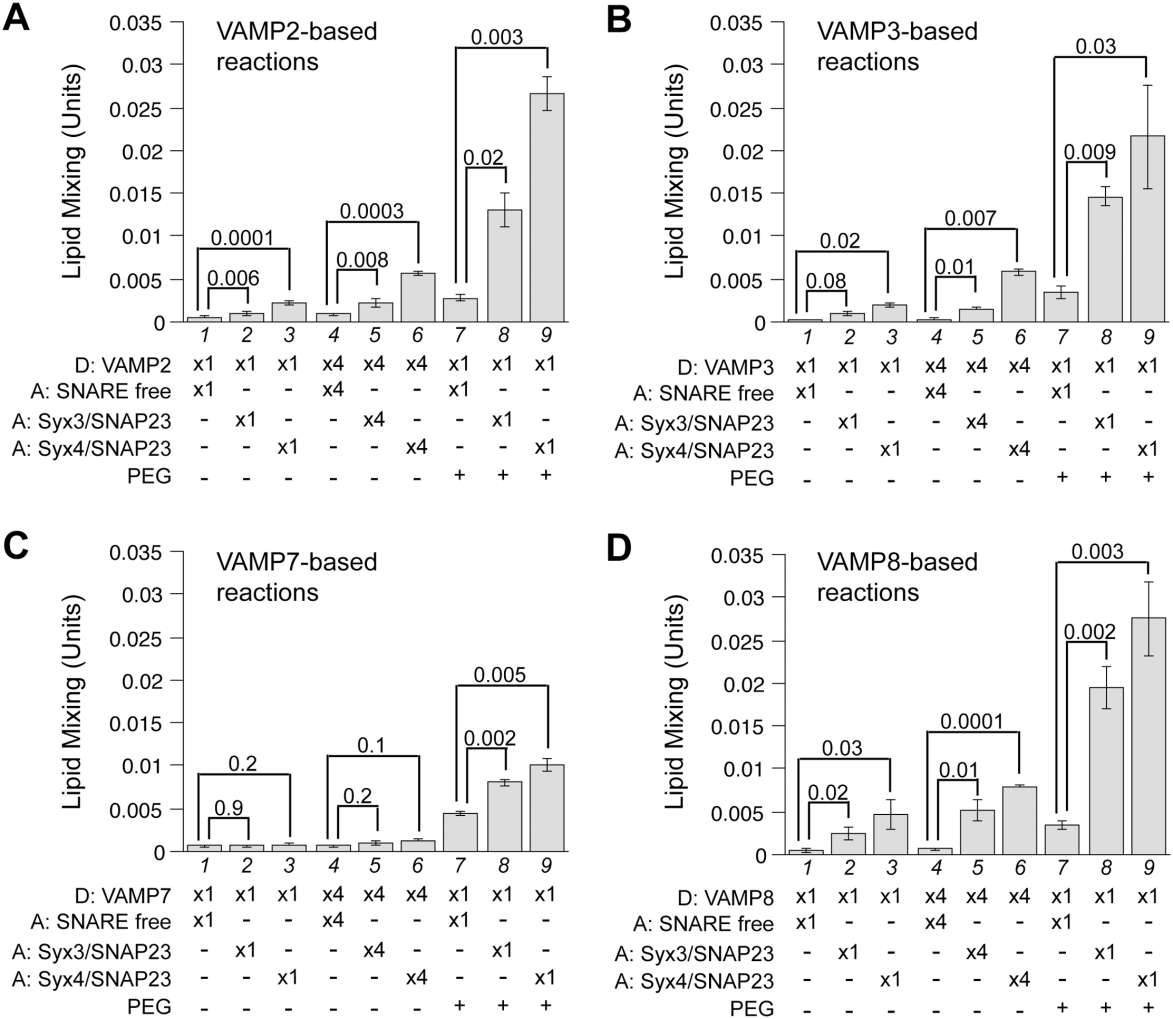
Functional pairing of SNAREs examined under enhanced tethering conditions. Donor RPLs bearing VAMP2 (A), VAMP3 (B), VAMP7 (C) or VAMP8 (D) were incubated with acceptor RPLs in standard fusion reactions (lanes 1 to 3), 4 x reactions (lanes 4 to 6), where the concentrations of the donor and acceptor RPLs were increased 4 fold to 200mM and 1600mM respectively. Fusion reactions including 4% PEG6000 (v/v) are presented in lanes 7 to 9. The maximal early rates of dequenching/lipid mixing were calculated as described in Materials and Methods. The mean values are presented and error bars represent standard deviations from at least three independent experiments. Where appropriate, p values calculated using Student’s t test are presented.

In a systematic effort to identify the putative trans-SNARE complexes in mast cell exocytosis, we purified all seven SNAREs (VAMP2, 3, 7, 8, syntaxin3, 4, and SNAP-23) that are either biochemically or functionally implicated in the degranulation process. As shown in Fig 1A, syntaxin3 and syntaxin4 (lanes 5 and 6) were incorporated along with SNAP-23 into the acceptor proteoliposomes (without NBD-DPPE or Rh-DPPE), whereas comparable amounts of VAMP2, 3, 7, 8 (lanes 1 to 4) were incorporated into the donor proteoliposomes, with NBD-DPPE and Rh-DPPE at quenching concentrations. Upon fusion, the mixture of the donor and acceptor membranes relieves this quenching effect via dilution, leading to increased NBD fluorescence [39]. The fluorescent signal was recorded in a plate reader every minute (Fig 1B to 1E). The maximal, early rate of dequenching was calculated (see Materials and Methods) to more effectively compare lipid mixing from different reaction conditions (Fig 2). In our standard assay, low but detectable levels of lipid mixing were observed in six combinations (Figs 1B to 1E and 2, lanes 1 to 3): i) VAMP2/syntaxin3/SNAP-23, ii) VAMP2/syntaxin4/SNAP-23, iii) VAMP3/syntaxin3/SNAP-23, iv) VAMP3/syntaxin4/SNAP-23, v) VAMP8/syntaxin3/SNAP-23, and vi) VAMP8/syntaxin4/SNAP-23. VAMP8 appears to be the most potent R-SNARE whereas VAMP7 shows no activity.

In eukaryotic cells, trans-SNARE pairing is facilitated by tethering factors (e.g., HOPS for vacuole fusion and the exocyst for neurotransmission), which bring vesicles/membranes into close proximity. This can be mimicked *in vitro* by increasing the concentrations of SNARE-bearing liposomes or by introducing synthetic polymer polyethylene glycol (e.g., PEG6000) [38,40]. Enhancing the concentrations of SNARE-bearing liposomes enhances the rate of the specific interaction between cognate SNAREs located on the donor and the acceptor, promoting their tethering and docking. PEG on the other hand induces nonspecific membrane tethering, which has been exploited extensively in previous studies of reconstituted SNARE-bearing proteoliposomes [41,42]. As shown in Fig 2, except for VAMP7, a 4-fold increase of both donor and acceptor liposomes modestly increased the rates of SNARE-mediated lipid mixing (compare lanes 2 and 5, 3 and 6). More drastic effects were observed when PEG was administered (lanes 8 and 9). Notably, the lipid mixing mediated by VAMP2/syntaxin3/SNAP-23 or VAMP3/syntaxin3/SNAP-23 that is almost negligible under standard reaction conditions now becomes readily detectable (Fig 2A and 2B, compare lanes 8 and 2). Even for VAMP7, Q-SNARE-dependent lipid mixing can be observed relative to the control (Fig 2C, compare lanes 8 and 9 to 7). These data indicate that an accurate assessment of trans-SNARE pairing in reconstitution may require conditions where the efficiency of tethering is optimized.

### Recapitulated SNARE-Dependent Mast Cell Exocytic Fusion Is Sensitive to SNAP and NSF

**Fig 3:**
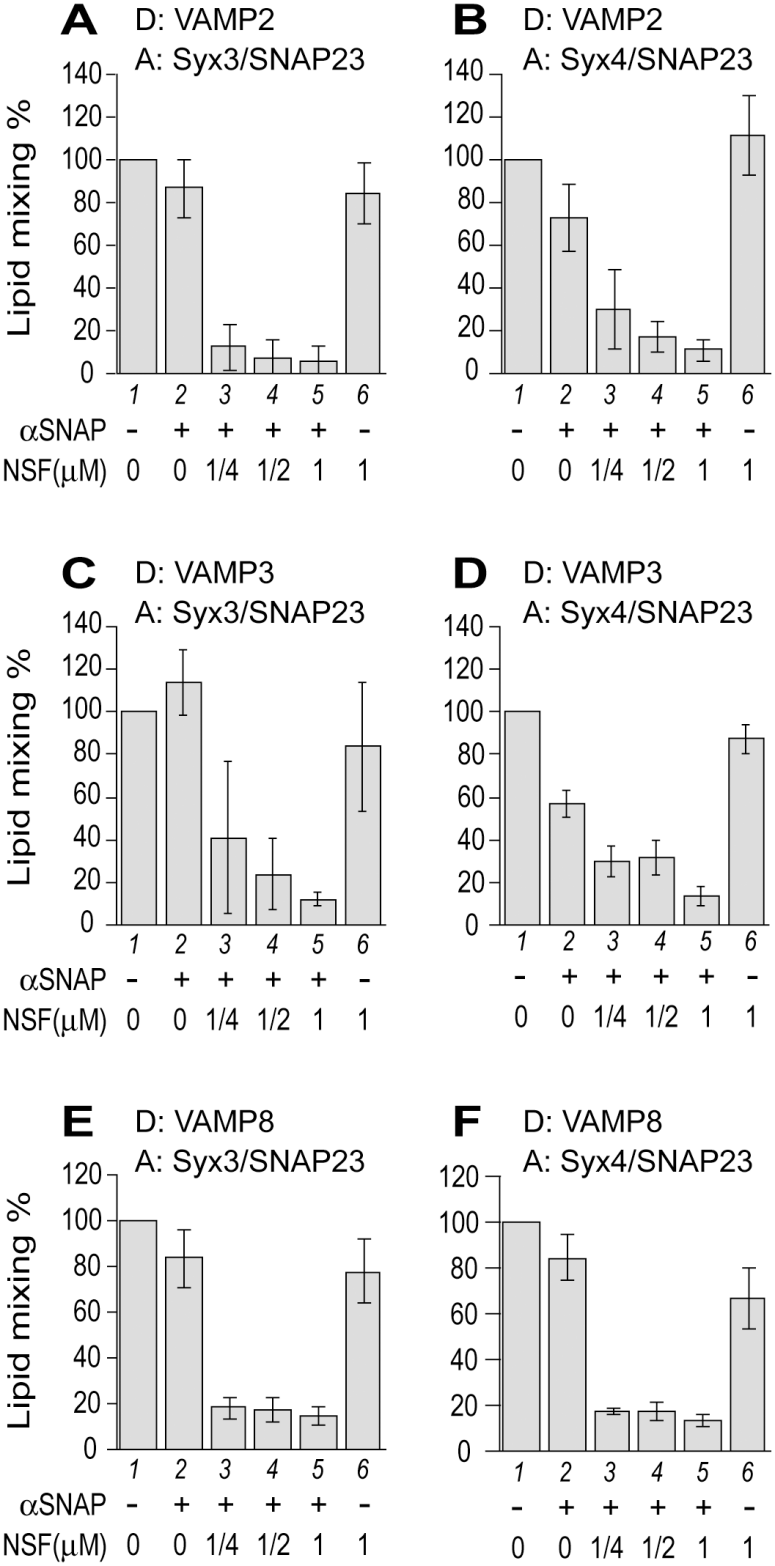
αSNAP/NSF-dependent inhibition of SNARE-mediated lipid mixing. (A and B) VAMP2-bearing donor RPLs were incubated with specified acceptor RPLs in fusion reactions containing 0.5mM ATP, 0.5mM MgCl_2_, and 4% PEG6000. Also included in the reactions are specified amounts of NSF along with 0.5μM αSNAP (A) or 0.15μM αSNAP (B). (C and D) VAMP3-bearing donor RPLs were incubated with specified acceptor RPLs in fusion reactions containing 1mM ATP, 1mM MgCl_2_, and 4% PEG6000. Also included in the reactions are specified amounts of NSF along with 0.15μM αSNAP. (E and F) VAMP8-bearing donor RPLs were incubated with specified acceptor RPLs in standard reactions containing 0.5mM ATP and 0.5mM MgCl_2_. Also included in the reactions are specified amounts of NSF along with 0.1μM αSNAP (E) or 0.02μM αSNAP (F). All samples contained the same amounts of αSNAP buffer and NSF buffer. The maximal early rates of lipid mixing for the SNARE-only reactions were used to generate the “standard” value (the lipid-mixing rate from SNARE-free RPLs was treated as a background and subtracted) and set as 100%. The values for other conditions were adjusted relative to the “standard” value. Error bars represent standard deviations from three independent experiments.

We went on to examine the nature of lipid mixing by introducing SNARE disassembly chaperones (αSNAP and NSF) into all except the VAMP7-based reactions. This is because the subdued VAMP7 activity—likely due to its inhibitory N-terminal longin domain [43]–prevents accurate assessment of αSNAP /NSF-dependent inhibition. Even in the presence of PEG, the fold change between the signal (Fig 2C, lanes 8 and 9) and the background (lane 7) would be too small for such investigation. For the other R-SNAREs (e.g., VAMP2, VAMP3, and VAMP8), the addition of αSNAP at high concentrations (S1 Fig) clearly inhibits reconstituted lipid-mixing reactions, which reflects αSNAP’s ability to prevent membrane fusion by binding to individual SNAREs, its *bona fide* receptors, or to the partially assembled trans-SNARE complex, as observed in other membrane fusion systems [44,45,46]. At non- or sub- inhibitory concentrations of αSNAP, addition of NSF, an ATPase, diminished the rates of lipid mixing (Fig 3). NSF by itself had minimal effect on most fusion reactions (Fig 3, compare lanes 1 and 6). Therefore, the cooperation of αSNAP and NSF is required to disassemble either the trans-SNARE complexes or the Q-SNARE subcomplexes prior to trans-SNARE zippering. Taken together, we conclude that all six sets of fusion reactions reconstituted in this study are underpinned by authentic trans-SNARE interactions.

### Munc18a Promotes the Lipid Mixing Mediated by Four Sets of SNAREs

**Fig 4:**
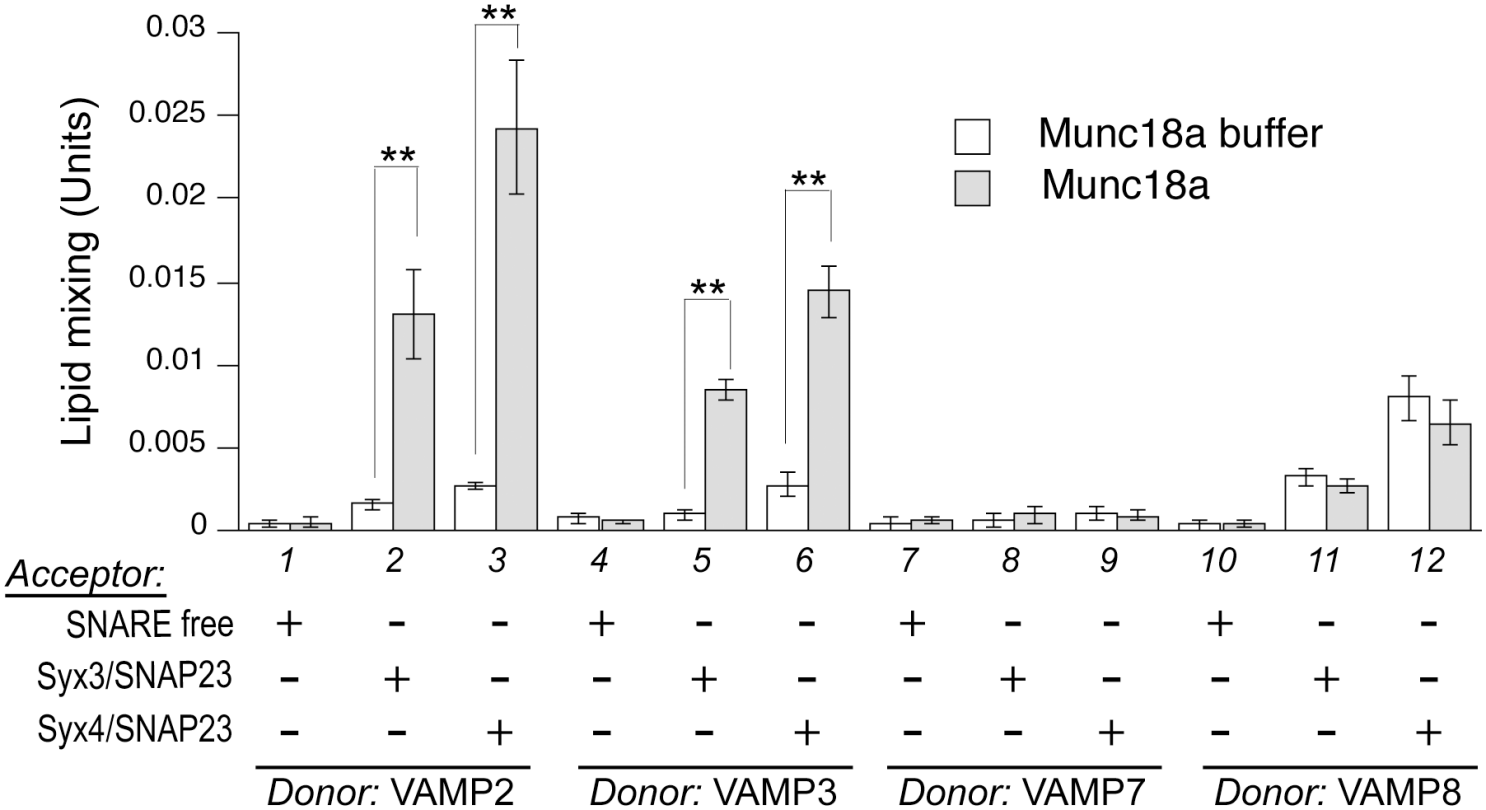
Munc18a selectively regulates different trans-SNARE complexes. Various combinations of donor and acceptor RPLs as specified were incubated overnight at 4°C with Munc18a (2μM) or control buffer, before transferring to 37°C. Error bars represent standard deviations from three independent experiments. p values were calculated using Student’s t test. ** indicates p < 0.01.

To investigate if Munc18a, b, and c operate through any of the trans-SNARE complexes we have identified, we purified recombinant forms of these proteins either from *E*. *coli* lysates or from cultured Sf9 cells (recombinant Munc18b and Munc18c expressed in *E*.*coli* are largely insoluble and difficult to purify). The N-terminal tags are readily removed by Tev protease (S2 Fig). When we initially tested Munc18a in lipid-mixing assays, it potently stimulated the rate of VAMP2-mediated lipid mixing in a concentration-dependent fashion (S3 Fig). At the suboptimal level (*e*.*g*., 2μM), it stimulated the lipid mixing mediated by VAMP2 (Fig 4, lanes 1 to 3), VAMP3 (lanes 4 to 6), but not by VAMP7 (lanes 7 to 9) or VAMP8 (lanes 10 to 12). The specificity of Munc18a for VAMP2 mirrors what had been previously observed in SNAP-25-mediated neurotransmission, where selectively replacing VAMP2 residues in the SNARE domain with corresponding residues in VAMP8 decreased Munc18a stimulation *in vitro* and reduced exocytosis *in vivo* [26]. Munc18a does not have any effect unless acceptor liposomes bear either syntaxin3/SNAP-23 or syntaxin4/SNAP-23, suggesting the presence of cognate Q-SNAREs on the apposing membrane is important for Munc18a action.

Intriguingly, neither Munc18b nor Munc18c exhibits positive effects in the fusion reactions (S4A and S4B Fig) as we had expected. The pull-down assay shows that Munc18c interacted with full-length syntaxin4 but not syntaxin3 (S4C Fig), which corroborates with published studies [31,47]. This interaction might be necessary for Munc18c-dependent inhibition of lipid mixing catalyzed by VAMP8/syntaxin4/SNAP-23 (S4B Fig, lane 12; S5D Fig), but does not appear to be the exclusive underlying mechanism since VAMP3-mediated lipid mixing was not negatively affected (S4B Fig, lane 6). In contrast to Munc18c, Munc18b had little effect on virtually all combinations tested. In addition to its reported ability to bind directly to syntaxin3 cytoplasmic domain [47], Munc18b also appears to bind membrane-anchored full-length syntaxin3 and syntaxin4 (S4C Fig). The significance of Munc18b/syntaxin4 interaction in mast cell exocytosis and membrane fusion requires future investigation.

Because Munc18s could regulate exocytosis via reversible interaction with the N-peptide in cognate syntaxins [48,49], we wished to address the concern whether the tetrapeptide (GAMG) remaining at the N-terminus of syntaxin4 after Tev cleavage might interfere with Munc18 function. We acquired an untagged syntaxin4 construct and co-expressed it along with His_6_-SNAP-23 as previously described [36]. Acceptor RPLs bearing untagged syntaxin4 behaved similarly to tagged syntaxin4, in their response to Munc18 isoforms (S5 Fig), suggesting that the extra N-terminal tetrapeptide had caused minimal impact in our assay. Based on reported studies in other secretory events [34,50], we propose that Munc18b or Munc18c might receive post-translational modifications in activated mast cells in order to promote SNARE-dependent granule exocytosis (see discussion).

### Munc18a-Dependent Stimulation Is Sensitive to Soluble Fragments of R-SNAREs at an Early Stage

**Fig 5:**
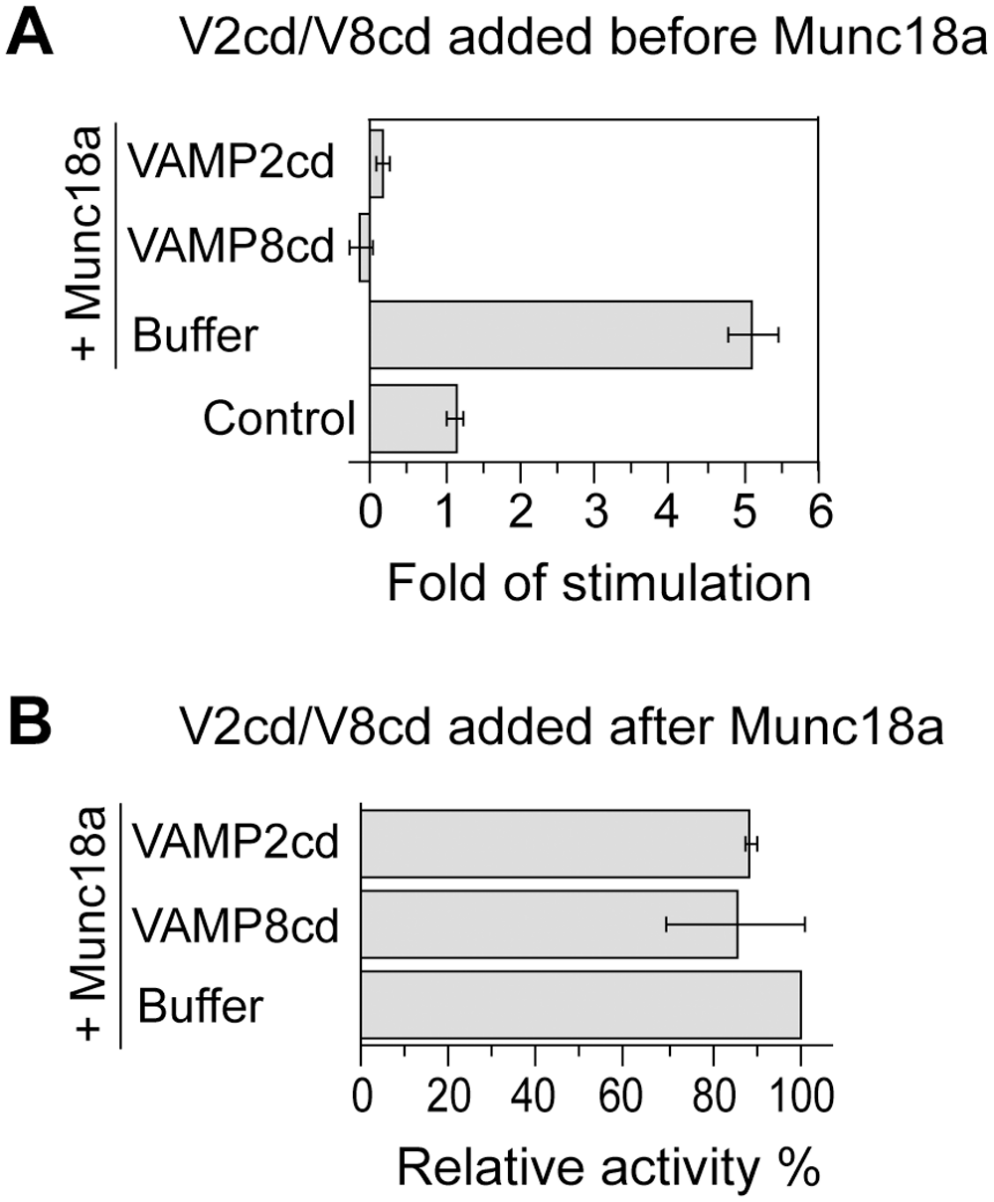
Munc18a-dependent stimulation is sensitive to inhibitory proteins at an early stage of the fusion reaction. (A). Acceptor RPLs bearing untagged syntaxin4/His_6_-SNAP-23 and VAMP2-bearing donor RPLs were incubated with inhibitory proteins VAMP2cd (2μM) or VAMP8cd (2μM) or buffer on ice overnight and then received 5μM Munc18a or MBP (control). The incubation was continued on ice for another 90 min before shifting to 37°C. Fold increases in the initial lipid-mixing rates of the fusion reactions are shown. In (B), the same RPLs were incubated first with 5μM Munc18a on ice for 90 min before the addition of VAMP2cd (2μM) or VAMP8cd (2μM). Following overnight incubation on ice, samples were transferred to 37°C to monitor NBD fluorescence. The maximal early rates of lipid mixing for the Munc18a-only reactions were used to generate the “standard” value and set as 100%. The values for other conditions were adjusted relative to the “standard” value. Error bars represent standard deviations from three independent experiments.

To further characterize the synergistic effect between Munc18a and VAMP2/syntaxin4/SNAP-23 in lipid mixing, we introduced the cytoplasmic domains of VAMP8 and VAMP2 at a concentration 8 fold of the full-length VAMP2 on the donor liposomes. These inhibitory proteins prevent SNARE-dependent fusion by competing for cognate Q-SNAREs [19,51]. When they were incubated with RPLs overnight on ice before the addition of Munc18a, very little lipid-mixing activities were observed (Fig 5A), showing that Munc18a-promoted lipid mixing requires functional trans-SNARE pairing. Intriguingly, when these inhibitory proteins were introduced to the reaction mixture 90 min after Munc18a addition, and the mixture was then incubated on ice overnight, there was very little inhibition (Fig 5B). These observations indicate that VAMP8cd or VAMP2cd does not poison the lipid-mixing reaction in any unspecific fashion. Rather, they act in a particular stage in the fusion cascade that is kinetically earlier than Munc18a action. We suggest that Munc18a either promotes the partial zippering of the trans-SNARE complex on ice, which becomes inaccessible to VAMP2cd or VAMP8cd, or the binding of Munc18a to the SNAREs prevents the access of the inhibitory proteins. Future studies are needed to distinguish these scenarios.

## Discussion

Identification of functional trans-SNARE complexes in mast cell exocytosis is often challenged by the presence of both compound degranulation and piecemeal degranulation, which can be further compounded by the heterogeneity of granule population inside the cell [7,15]. Nevertheless, in addition to VAMP8/syntaxin4/SNAP-23, biochemical characterizations, cellular localization studies, and cell-based functional analysis have collectively indicated the requirement of syntaxin3-based trans-SNARE complexes in mast cell exocytosis [9,16,20,52]. Our reconstitution of several fusogenic syntaxin3-containing trans-SNARE complexes under enhanced tethering conditions lends strong support to the notion that distinct trans-SNARE complexes underscore various types of granule/lysosome exocytosis in mast cells.

All the SNARE-only fusion reactions tested in this study are sensitive to action of ATPase NSF and its cofactor αSNAP. This is consistent with the reported behavior of proteoliposomes bearing vacuolar SNAREs, which do not fuse at all in the presence of Sec17p (αSNAP homolog in yeast) and Sec18p (NSF homolog in yeast) unless the HOPS tethering complex is also present [38] [53]. Intriguingly, the reconstituted synaptic vesicle fusion appears to resist the disassembly effects of αSNAP and NSF [54]. In the absence of a self-inhibitory domain of syntaxin1A, the addition of αSNAP and NSF could even accelerate the lipid mixing. It is conceivable that, due to the differences in the SNARE complexes or the different lipid composition in different proteoliposome fusion systems, the neuronal trans-SNARE complex executes fusion at rate faster than the rate of turnover by αSNAP and NSF [53].

How could Munc18a promote β-hexosaminidase release [30] if it does not operate through VAMP8-based trans-SNARE complexes (Fig 4, lanes 11 and 12)? Our observation that Munc18a stimulates VAMP2- and VAMP3- dependent lipid mixing implies that a distinct set of trans-SNARE complex might form in the event that VAMP8-dependent degranulation is compromised. In agreement to this, a ternary complex of VAMP2, syntaxin4 and SNAP-23 was identified in the lipid rafts during mast cell exocytosis [55], and more importantly, increasing amounts of VAMP2 and VAMP3 were found in association with SNAP-23 in VAMP8-knockout cells [7,8]. Similar compensatory mechanisms have previously been observed in intracellular traffic. In baker’s yeast, a predominantly Golgi-localized SNARE Ykt6p is up-regulated in *sec22* deletion strains to sustain ER to Golgi traffic [56] and in *nyv1* deletion strains to sustain vacuolar fusion [57]. In animal cells, VAMP3 can partially compensate for the deletion of VAMP2 in calcium-triggered exocytosis [58,59]. Although blocking or knocking down VAMP2 or VAMP3 alone had limited effects on β-hexosaminidase release [10,19], we hypothesize that simultaneously knocking out VAMP2, VAMP3, and VAMP8 would abolish mast cell exocytosis.

Concerning the activators for VAMP8-based degranulation, Munc18 isoforms remain the top candidates. Although none of the three unmodified Munc18s operate synergistically with VAMP8-based trans-SNARE complexes in this study, a number of reports have documented the importance of reversible phosphorylation in Munc18 activity. For example, site-specific phosphorylation of Munc18a by PKC has been found critical for neurotransmission [60]. Since the PKC pathway is also active in mast cell degranulation [61,62,63], it will be interesting to examine if the same Munc18a modifications also takes place and whether they alter the specificity of Munc18a. Similarly, polarized secretion in epithelial cells requires CDK5-dependent phosphorylation of Munc18b at Thr572, which promotes the assembly of the functional Munc18b/VAMP2/syntaxin3/SNAP-25 membrane fusion machinery [50]. For GLUT4 exocytosis in fat and muscle cells, Munc18c phosphorylation at Y521 promotes SNARE complex formation between VAMP2, syntaxin4, and SNAP-23 [34], whereas the unmodified Munc18c inhibits membrane fusion via specific interaction with syntaxin4 [34,35]. Future studies of signaling-dependent modifications of Munc18 in activated mast cells will provide new insights into the selective regulation of degranulation-relevant trans-SNARE complexes.

## Supporting Information

S1 Fig Effects of αSNAP on SNARE-only reactions. (EPS)

S2 Fig Purified Munc18s are TEV cleavable. (EPS)

S3 Fig Munc18a stimulates VAMP2-mediated lipid mixing in a concentration dependent fashion. (EPS)

S4 Fig The effects of Munc18b and Munc18c in reconstituted fusion reactions. (EPS)

S5 Fig The extra tetrapeptide at the N-terminus of the recombinant syntaxin4 does not alter the selective activities of Munc18s. (EPS)

S1 File Supplemental Figure Legends. (DOCX)

S1 Table Oligonucleotide primers used in PCR for cloning. (PDF)

## References

[1] Wernersson S, Pejler G (2014) Mast cell secretory granules: armed for battle. Nat Rev Immunol 14: 478–494. 10.1038/nri3690 24903914

[2] Pickett JA, Edwardson JM (2006) Compound exocytosis: mechanisms and functional significance. Traffic 7: 109–116. 16420520 10.1111/j.1600-0854.2005.00372.x

[3] Crivellato E, Nico B, Gallo VP, Ribatti D (2010) Cell secretion mediated by granule-associated vesicle transport: a glimpse at evolution. Anat Rec (Hoboken) 293: 1115–1124. 20340095 10.1002/ar.21146

[4] Jahn R, Scheller RH (2006) SNAREs—engines for membrane fusion. Nat Rev Mol Cell Biol 7: 631–643. 16912714 10.1038/nrm2002

[5] Woska JR Jr, Gillespie ME (2011) Small-interfering RNA-mediated identification and regulation of the ternary SNARE complex mediating RBL-2H3 mast cell degranulation. Scand J Immunol 73: 8–17. 10.1111/j.1365-3083.2010.02471.x 21128998

[6] Paumet F, Le Mao J, Martin S, Galli T, David B, Blank U, et al (2000) Soluble NSF attachment protein receptors (SNAREs) in RBL-2H3 mast cells: functional role of syntaxin 4 in exocytosis and identification of a vesicle-associated membrane protein 8-containing secretory compartment. J Immunol 164: 5850–5857. 10820264 10.4049/jimmunol.164.11.5850

[7] Tiwari N, Wang CC, Brochetta C, Ke G, Vita F, Qi Z, et al (2008) VAMP-8 segregates mast cell-preformed mediator exocytosis from cytokine trafficking pathways. Blood 111: 3665–3674. 10.1182/blood-2007-07-103309 18203950

[8] Tiwari N, Wang CC, Brochetta C, Scandiuzzi L, Hong W, Blank U (2009) Increased formation of VAMP-3-containing SNARE complexes in mast cells from VAMP-8 deficient cells. Inflamm Res 58 Suppl 1: 13–14. 10.1007/s00011-009-0645-y 19274433

[9] Brochetta C, Suzuki R, Vita F, Soranzo MR, Claver J, Celia L, et al (2014) Munc18-2 and syntaxin 3 control distinct essential steps in mast cell degranulation. J Immunol 192: 41–51. 10.4049/jimmunol.1301277 24323579 PMC3905451

[10] Sander LE, Frank SP, Bolat S, Blank U, Galli T, Bigalke H, et al (2008) Vesicle associated membrane protein (VAMP)-7 and VAMP-8, but not VAMP-2 or VAMP-3, are required for activation-induced degranulation of mature human mast cells. Eur J Immunol 38: 855–863. 10.1002/eji.200737634 18253931

[11] Suzuki K, Verma IM (2008) Phosphorylation of SNAP-23 by IkappaB kinase 2 regulates mast cell degranulation. Cell 134: 485–495. 10.1016/j.cell.2008.05.050 18692471 PMC2586340

[12] Hibi T, Hirashima N, Nakanishi M (2000) Rat basophilic leukemia cells express syntaxin-3 and VAMP-7 in granule membranes. Biochem Biophys Res Commun 271: 36–41. 10777677 10.1006/bbrc.2000.2591

[13] Guo Z, Turner C, Castle D (1998) Relocation of the t-SNARE SNAP-23 from lamellipodia-like cell surface projections regulates compound exocytosis in mast cells. Cell 94: 537–548. 9727496 10.1016/s0092-8674(00)81594-9

[14] Puri N, Kruhlak MJ, Whiteheart SW, Roche PA (2003) Mast cell degranulation requires N-ethylmaleimide-sensitive factor-mediated SNARE disassembly. J Immunol 171: 5345–5352. 14607937 10.4049/jimmunol.171.10.5345

[15] Puri N, Roche PA (2008) Mast cells possess distinct secretory granule subsets whose exocytosis is regulated by different SNARE isoforms. Proc Natl Acad Sci U S A 105: 2580–2585. 10.1073/pnas.0707854105 18250339 PMC2268179

[16] Tadokoro S, Kurimoto T, Nakanishi M, Hirashima N (2007) Munc18-2 regulates exocytotic membrane fusion positively interacting with syntaxin-3 in RBL-2H3 cells. Mol Immunol 44: 3427–3433. 17408745 10.1016/j.molimm.2007.02.013

[17] Fasshauer D, Sutton RB, Brunger AT, Jahn R (1998) Conserved structural features of the synaptic fusion complex: SNARE proteins reclassified as Q- and R-SNAREs. Proc Natl Acad Sci U S A 95: 15781–15786. 9861047 10.1073/pnas.95.26.15781 PMC28121

[18] Lorentz A, Baumann A, Vitte J, Blank U (2012) The SNARE Machinery in Mast Cell Secretion. Front Immunol 3: 143 10.3389/fimmu.2012.00143 22679448 PMC3367400

[19] Lippert U, Ferrari DM, Jahn R (2007) Endobrevin/VAMP8 mediates exocytotic release of hexosaminidase from rat basophilic leukaemia cells. FEBS Lett 581: 3479–3484. 17618625 10.1016/j.febslet.2007.06.057

[20] Pombo I, Rivera J, Blank U (2003) Munc18-2/syntaxin3 complexes are spatially separated from syntaxin3-containing SNARE complexes. FEBS Lett 550: 144–148. 12935901 10.1016/s0014-5793(03)00864-0

[21] Yang Y, Oh JM, Heo P, Shin JY, Kong B, Shin J, et al (2013) Polyphenols differentially inhibit degranulation of distinct subsets of vesicles in mast cells by specific interaction with granule-type-dependent SNARE complexes. Biochem J 450: 537–546. 10.1042/BJ20121256 23252429 PMC4831212

[22] Sakiyama H, Tadokoro S, Nakanishi M, Hirashima N (2009) Membrane fusion between liposomes containing SNARE proteins involved in mast cell exocytosis. Inflamm Res 58: 139–142. 10.1007/s00011-008-7173-z 19109692

[23] Theoharides TC, Kops SK, Bondy PK, Askenase PW (1985) Differential release of serotonin without comparable histamine under diverse conditions in the rat mast cell. Biochem Pharmacol 34: 1389–1398. 2581583 10.1016/0006-2952(85)90675-6

[24] Sudhof TC, Rothman JE (2009) Membrane fusion: grappling with SNARE and SM proteins. Science 323: 474–477. 10.1126/science.1161748 19164740 PMC3736821

[25] Munson M, Bryant NJ (2009) A role for the syntaxin N-terminus. Biochem J 418: e1–3. 10.1042/BJ20082389 19159342 PMC5502781

[26] Shen J, Tareste DC, Paumet F, Rothman JE, Melia TJ (2007) Selective activation of cognate SNAREpins by Sec1/Munc18 proteins. Cell 128: 183–195. 17218264 10.1016/j.cell.2006.12.016

[27] Schraw TD, Lemons PP, Dean WL, Whiteheart SW (2003) A role for Sec1/Munc18 proteins in platelet exocytosis. Biochem J 374: 207–217. 12773094 10.1042/BJ20030610 PMC1223584

[28] Tellam JT, McIntosh S, James DE (1995) Molecular identification of two novel Munc-18 isoforms expressed in non-neuronal tissues. J Biol Chem 270: 5857–5863. 7890715 10.1074/jbc.270.11.5857

[29] Zhang W, Efanov A, Yang SN, Fried G, Kolare S, Brown H, et al (2000) Munc-18 associates with syntaxin and serves as a negative regulator of exocytosis in the pancreatic beta-cell. J Biol Chem 275: 41521–41527. 11024017 10.1074/jbc.M005479200

[30] Bin NR, Jung CH, Piggott C, Sugita S (2013) Crucial role of the hydrophobic pocket region of Munc18 protein in mast cell degranulation. Proc Natl Acad Sci U S A 110: 4610–4615. 10.1073/pnas.1214887110 23487749 PMC3607013

[31] Martin-Verdeaux S, Pombo I, Iannascoli B, Roa M, Varin-Blank N, Rivera J, et al (2003) Evidence of a role for Munc18-2 and microtubules in mast cell granule exocytosis. J Cell Sci 116: 325–334. 12482918 10.1242/jcs.00216

[32] Thurmond DC, Kanzaki M, Khan AH, Pessin JE (2000) Munc18c function is required for insulin-stimulated plasma membrane fusion of GLUT4 and insulin-responsive amino peptidase storage vesicles. Mol Cell Biol 20: 379–388. 10594040 10.1128/mcb.20.1.379-388.2000 PMC85093

[33] Kanda H, Tamori Y, Shinoda H, Yoshikawa M, Sakaue M, Udagawa J, et al (2005) Adipocytes from Munc18c-null mice show increased sensitivity to insulin-stimulated GLUT4 externalization. J Clin Invest 115: 291–301. 15690082 10.1172/JCI22681 PMC546422

[34] Kioumourtzoglou D, Gould GW, Bryant NJ (2014) Insulin stimulates syntaxin4 SNARE complex assembly via a novel regulatory mechanism. Mol Cell Biol 34: 1271–1279. 10.1128/MCB.01203-13 24469400 PMC3993566

[35] Brandie FM, Aran V, Verma A, McNew JA, Bryant NJ, Gould GW (2008) Negative regulation of syntaxin4/SNAP-23/VAMP2-mediated membrane fusion by Munc18c in vitro. PLoS One 3: e4074 10.1371/journal.pone.0004074 19116655 PMC2605266

[36] Yu H, Rathore SS, Lopez JA, Davis EM, James DE, Martin JL, et al (2013) Comparative studies of Munc18c and Munc18-1 reveal conserved and divergent mechanisms of Sec1/Munc18 proteins. Proc Natl Acad Sci U S A 110: E3271–3280. 10.1073/pnas.1311232110 23918365 PMC3761595

[37] Sheffield P, Garrard S, Derewenda Z (1999) Overcoming expression and purification problems of RhoGDI using a family of "parallel" expression vectors. Protein Expr Purif 15: 34–39. 10024467 10.1006/prep.1998.1003

[38] Mima J, Hickey CM, Xu H, Jun Y, Wickner W (2008) Reconstituted membrane fusion requires regulatory lipids, SNAREs and synergistic SNARE chaperones. EMBO J 27: 2031–2042. 10.1038/emboj.2008.139 18650938 PMC2516887

[39] Struck DK, Hoekstra D, Pagano RE (1981) Use of resonance energy transfer to monitor membrane fusion. Biochemistry 20: 4093–4099. 7284312 10.1021/bi00517a023

[40] Furukawa N, Mima J (2014) Multiple and distinct strategies of yeast SNAREs to confer the specificity of membrane fusion. Sci Rep 4: 4277 10.1038/srep04277 24589832 PMC3940976

[41] Dennison SM, Bowen ME, Brunger AT, Lentz BR (2006) Neuronal SNAREs do not trigger fusion between synthetic membranes but do promote PEG-mediated membrane fusion. Biophys J 90: 1661–1675. 16339880 10.1529/biophysj.105.069617 PMC1367317

[42] Hickey CM, Wickner W (2010) HOPS initiates vacuole docking by tethering membranes before trans-SNARE complex assembly. Mol Biol Cell 21: 2297–2305. 10.1091/mbc.E10-01-0044 20462954 PMC2893992

[43] Burgo A, Casano AM, Kuster A, Arold ST, Wang G, Nola S, et al (2013) Increased activity of the vesicular soluble N-ethylmaleimide-sensitive factor attachment protein receptor TI-VAMP/VAMP7 by tyrosine phosphorylation in the Longin domain. J Biol Chem 288: 11960–11972. 10.1074/jbc.M112.415075 23471971 PMC3636883

[44] Woodman PG (1997) The roles of NSF, SNAPs and SNAREs during membrane fusion. Biochim Biophys Acta 1357: 155–172. 9223620 10.1016/s0167-4889(97)00039-6

[45] Park Y, Vennekate W, Yavuz H, Preobraschenski J, Hernandez JM, Riedel D, et al (2014) alpha-SNAP Interferes with the Zippering of the SNARE Protein Membrane Fusion Machinery. J Biol Chem 289: 16326–16335. 10.1074/jbc.M114.556803 24778182 PMC4047401

[46] Wang L, Ungermann C, Wickner W (2000) The docking of primed vacuoles can be reversibly arrested by excess Sec17p (alpha-SNAP). J Biol Chem 275: 22862–22867. 10816559 10.1074/jbc.M001447200

[47] Peng RW, Guetg C, Abellan E, Fussenegger M (2010) Munc18b regulates core SNARE complex assembly and constitutive exocytosis by interacting with the N-peptide and the closed-conformation C-terminus of syntaxin 3. Biochem J 431: 353–361. 10.1042/BJ20100145 20695848

[48] Hu SH, Latham CF, Gee CL, James DE, Martin JL (2007) Structure of the Munc18c/Syntaxin4 N-peptide complex defines universal features of the N-peptide binding mode of Sec1/Munc18 proteins. Proc Natl Acad Sci U S A 104: 8773–8778. 17517664 10.1073/pnas.0701124104 PMC1885578

[49] Rathore SS, Bend EG, Yu H, Hammarlund M, Jorgensen EM, Shen J (2010) Syntaxin N-terminal peptide motif is an initiation factor for the assembly of the SNARE-Sec1/Munc18 membrane fusion complex. Proc Natl Acad Sci U S A 107: 22399–22406. 10.1073/pnas.1012997108 21139055 PMC3012463

[50] Liu Y, Ding X, Wang D, Deng H, Feng M, Wang M, et al (2007) A mechanism of Munc18b-syntaxin 3-SNAP25 complex assembly in regulated epithelial secretion. FEBS Lett 581: 4318–4324. 17716669 10.1016/j.febslet.2007.07.083 PMC3690314

[51] Weber T, Zemelman BV, McNew JA, Westermann B, Gmachl M, Parlati F, et al (1998) SNAREpins: minimal machinery for membrane fusion. Cell 92: 759–772. 9529252 10.1016/s0092-8674(00)81404-x

[52] Frank SP, Thon KP, Bischoff SC, Lorentz A (2011) SNAP-23 and syntaxin-3 are required for chemokine release by mature human mast cells. Mol Immunol 49: 353–358. 10.1016/j.molimm.2011.09.011 21981832

[53] Xu H, Jun Y, Thompson J, Yates J, Wickner W (2010) HOPS prevents the disassembly of trans-SNARE complexes by Sec17p/Sec18p during membrane fusion. EMBO J 29: 1948–1960. 10.1038/emboj.2010.97 20473271 PMC2892374

[54] Weber T, Parlati F, McNew JA, Johnston RJ, Westermann B, Sollner TH, et al (2000) SNAREpins are functionally resistant to disruption by NSF and alphaSNAP. J Cell Biol 149: 1063–1072. 10831610 10.1083/jcb.149.5.1063 PMC2174819

[55] Puri N, Roche PA (2006) Ternary SNARE complexes are enriched in lipid rafts during mast cell exocytosis. Traffic 7: 1482–1494. 16984405 10.1111/j.1600-0854.2006.00490.x

[56] Liu Y, Barlowe C (2002) Analysis of Sec22p in endoplasmic reticulum/Golgi transport reveals cellular redundancy in SNARE protein function. Mol Biol Cell 13: 3314–3324. 12221135 10.1091/mbc.E02-04-0204 PMC124161

[57] Thorngren N, Collins KM, Fratti RA, Wickner W, Merz AJ (2004) A soluble SNARE drives rapid docking, bypassing ATP and Sec17/18p for vacuole fusion. EMBO J 23: 2765–2776. 15241469 10.1038/sj.emboj.7600286 PMC514947

[58] Bhattacharya S, Stewart BA, Niemeyer BA, Burgess RW, McCabe BD, Lin P, et al (2002) Members of the synaptobrevin/vesicle-associated membrane protein (VAMP) family in Drosophila are functionally interchangeable in vivo for neurotransmitter release and cell viability. Proc Natl Acad Sci U S A 99: 13867–13872. 12364587 10.1073/pnas.202335999 PMC129789

[59] Borisovska M, Zhao Y, Tsytsyura Y, Glyvuk N, Takamori S, Matti U, et al (2005) v-SNAREs control exocytosis of vesicles from priming to fusion. EMBO J 24: 2114–2126. 15920476 10.1038/sj.emboj.7600696 PMC1150890

[60] Genc O, Kochubey O, Toonen RF, Verhage M, Schneggenburger R (2014) Munc18-1 is a dynamically regulated PKC target during short-term enhancement of transmitter release. Elife 3: e01715 10.7554/eLife.01715 24520164 PMC3919271

[61] Chakravarty N, Kjeldsen B, Hansen M, Nielsen EH (1990) The involvement of protein kinase C in exocytosis in mast cells. Exp Cell Res 186: 245–249. 1688804 10.1016/0014-4827(90)90302-q

[62] Koopmann WR Jr, Jackson RC (1990) Calcium- and guanine-nucleotide-dependent exocytosis in permeabilized rat mast cells. Modulation by protein kinase C. Biochem J 265: 365–373. 1689146 10.1042/bj2650365 PMC1136896

[63] Pernas-Sueiras O, Alfonso A, Vieytes MR, Botana LM (2006) PKC and cAMP positively modulate alkaline-induced exocytosis in the human mast cell line HMC-1. J Cell Biochem 99: 1651–1663. 16823786 10.1002/jcb.21009

