# Supplementary material for: Differential Effects of Munc18s on Multiple Degranulation-Relevant Trans-SNARE Complexes
Source: PLoS One. 2015 Sep 18;10(9):e0138683. doi: 10.1371/journal.pone.0138683 (PMC4575180; doi:10.1371/journal.pone.0138683)
Supplement: S1 File — (DOCX) [file pone.0138683.s006.docx]

**S1 File. Supplemental Figure Legends**

**S1 Fig. Effects of αSNAP on SNARE-only reactions**. (A to F) VAMP2-, VAMP3-, and VAMP8- bearing donor RPLs were incubated with specified acceptor RPLs in standard reactions including 0.5mM ATP, 0.5mM MgCl_2_, and αSNAP (0μM, 0.017μM, 0.05μM, 0.15μM, 0.45μM, 1.3μM, 4μM, 12μM), with (A to D) or without 4% PEG6000 (E and F). All tubes contained the same amount of αSNAP buffer. The maximal rates of lipid mixing for the SNARE-only reactions were used to generate the “standard” value (the lipid-mixing rate from SNARE-free RPLs was treated as a background and subtracted) and set as 100%. The values for other conditions were adjusted relative to the “standard” value.

**S2 Fig. Purified recombinant Munc18s are TEV cleavable.** Two μM of MBP-Munc18a, His_6_-Munc18b, or His_6_-Munc18c were incubated with 1 μM His_6_-Tev or buffer at 4 °C overnight before 12μL of each reaction mixture were subject to SDS-PAGE and Coomassie-blue staining. Protein standard was loaded in the first lane. Arrow points to the position of His_6_-Tev.

**S3 Fig.** **Munc18a stimulates VAMP2-mediated lipid mixing in a concentration-dependent fashion**. Proteoliposomes as specified were incubated with increasing amounts of Munc18a at 4 °C overnight. Lipid mixing was subsequently measured at 37 °C. All samples contained the same amount of Munc18a buffer.

**S4 Fig. The effects of Munc18b and Munc18c in reconstituted fusion reactions.** Various combinations of donor and acceptor RPLs as specified were incubated overnight at 4 °C with either (A) Munc18b (5μM), (B) Munc18c (3μM) or their respective buffers, before transferring to 37 °C. Munc18c buffer contains 0.25mM DTT, which increased the rates of SNARE-based lipid mixing. Error bars represent standard deviations from three independent experiments. **p values were calculated using Student’s t test. ** indicates p < 0.01 and * indicates p < 0.05.** (C) RPLs (2.25mM lipids) bearing syntaxin3 or syntaxin4 were incubated with His_6_-Munc18s (2.5μM) in a volume of 20μL on ice for 100 min before 200μL of RB150 containing 1% Triton X-100 and 20mM imidazole were added. Pre-equilibrated Ni^+^ resin was immediately used to pull down His_6_-tagged Munc18 and associating proteins from the detergent lysates. Pull-downs were subject to SDS-PAGE followed by Coomassie-blue staining.

**S5 Fig.** **The extra tetrapeptide at the N-terminus of the recombinant syntaxin4 does not alter the selective activities of Munc18s**. Acceptor RPLs bearing untagged syntaxin4/His_6_-SNAP-23 were incubated with (A) VAMP2-bearing, (B) VAMP3-bearing, (C) VAMP7-bearing, and (D) VAMP8-bearing donor RPLs on ice overnight. Munc18 isoforms (5μM) (or buffer) were then added as specified. Following further incubation on ice for 90 min, lipid mixing was measured at 37 °C. All reactions contained 1mM of DTT.
