# Supplementary material for: Differential Effects of Munc18s on Multiple Degranulation-Relevant Trans-SNARE Complexes
Source: PLoS One. 2015 Sep 18;10(9):e0138683. doi: 10.1371/journal.pone.0138683 (PMC4575180; doi:10.1371/journal.pone.0138683)
Supplement: S1 Table — (PDF) [file pone.0138683.s007.pdf]

S1 Table. Oligonucleotide primers used in PCR for cloning.

Gene	Primer Sequence*	Lab ID
SNAP-23 (rat)	5'- tgccatgggaATGGATGATCTATCACCAG	HXO-C05
	5'- cggaattcTTAGCTGTCAATGAGTTTCTTTGC	HXO-C06
Syntaxin3 (rat)	5'- tgccatgggaATGAAGGACCGACTGGAG	HXO-C01
	5'- cggaattcTTATTTCAGCCCAACGGAC	HXO-C02
Syntaxin4 (rat)	5'- tgccatgggaATGCGCGACAGGACCC	HXO-C03
	5'- cggaattcTTATCCAACGGTTATGGTG	HXO-C04
VAMP2 (rat)	5'- tgccatgggaATGTCGGCTACCGCTGC	HXO-C11
	5'- cggaattcTTAAGTGCTGAAGTAAACGATG	HXO-C12
VAMP2cd (rat)	5'- tgccatgggaATGTCGGCTACCGCTGC	HXO-C11
	5'- gcgaattcTTACTTGAGGTTTTTCCACC	HXO-C71
VAMP3 (rat)	5'- aaaccatgggcATGTCTACAGGGGTGCCTTC	HXO-C69
	5'- gcgaattcTTAAGAGACACACCACACAATG	HXO-C70
VAMP7 (rat)	5'- *TTTAAGAAGGAGATATAGTTC*ATGGCCATTCTTTTTGCCGTT	HXO-C73
	5'- *GGATTGGAAGTAGAGGTTCTC*TTTCTTCACACAGCTTGGCCA	HXO-C74
VAMP8 (rat)	5'- tgccATGGAGGCCAGTGGGAG	HXO-C09
	5'- cggaattcTTAAGTGGGGATGGTGC	HXO-C10
VAMP8cd (rat)	5'- tgccATGGAGGCCAGTGGGAG	HXO-C09
	5'- gcgaattcTTACATCTTCACATTCTTCCACC	HXO-C72
Munc18a (rat)	5'- cggaattcATGGCCCCCATTGGCC	HXO-C43
	5'- acgcgtcgacTTAACTGCTTATTTCTTCGTCTG	HXO-C44
Munc18b (rat)	5'- cggaattcATGGCGCCCTTGGGGC	HXO-C45
	5'- CCTGTGGTCTCATACCTGTATG	HXO-C64
	5'- CATACAGGTATGAGACCACAGG	HXO-C30
	5'- acgcgtcgacTCAGGGCAGGGCTATGTCCTC	HXO-C46
	5'- atgggatccATGGCGCCCTTGGGGC	HXO-C67

*The start codon of each gene is in bold. Restriction sites used for cloning are underlined.
